# A 65-Day Fumonisin B Exposure at High Dietary Levels Has Negligible Effects on the Testicular and Spermatological Parameters of Adult Rabbit Bucks

**DOI:** 10.3390/toxins13040237

**Published:** 2021-03-25

**Authors:** András Szabó, Szabolcs Nagy, Omeralfaroug Ali, Zsolt Gerencsér, Miklós Mézes, Krisztián Milán Balogh, Tibor Bartók, Levente Horváth, Aziz Mouhanna, Melinda Kovács

**Affiliations:** 1 MTA-KE-SZIE Mycotoxins in the Food Chain Research Group, Department of Physiology and Animal Health, Institute of Physiology and Nutrition, Kaposvár Campus, Hungarian University of Agriculture and Life Sciences, Guba S. u. 40., 7400 Kaposvár, Hungary; kovacs.melinda@uni-mate.hu; 2 Department of Physiology and Animal Health, Institute of Physiology and Nutrition, Kaposvár Campus, Hungarian University of Agriculture and Life Sciences, Guba S. u. 40., 7400 Kaposvár, Hungary; omeralfaroug.ali@gmail.com (O.A.); aziz.mouhanna.1993.2@gmail.com (A.M.); 3 Department of Precision Livestock Farming and Animal Biotechnics, Institute of Animal Sciences, Georgikon Campus, Hungarian University of Agriculture and Life Sciences, Deák F. u. 16., 8360 Keszthely, Hungary; nagy.szabolcs.tamas@uni-mate.hu; 4 Department of Animal Breeding, Institute of Animal Sciences, Kaposvár Campus, Hungarian University of Agriculture and Life Sciences, Guba S. u. 40., 7400 Kaposvár, Hungary; Gerencser.zsolt@uni-mate.hu; 5 Department of Feed Toxicology, Institute of Physiology and Nutrition, Gödöllő Campus, Hungarian University of Agriculture and Life Sciences, Páter K. u. 1., 2053 Gödöllő, Hungary; mezes.miklos@uni-mate.hu (M.M.); balogh.krisztian.milan@uni-mate.hu (K.M.B.); 6 Fumizol Ltd., Kisfaludy u. 6/b, 6725 Szeged, Hungary; tibor.bartok@fumizol.hu (T.B.); levente.horvath89@gmail.com (L.H.)

**Keywords:** fumonisin B series, rabbit, testis, spermatozoa, membrane fatty acids, oxidative stress

## Abstract

A 65-day study was undertaken to test the effects of two doses (10 and 20 mg/kg) of dietary fumonisin Bs (FB) on the rabbit male reproduction system. Body and testicular weight was not affected by the intoxication, neither the fatty acid composition of the testicular total phospholipids; the testis histological analysis failed to reveal any toxic effect. The FBs increased the testicular concentration and activity of reduced glutathione and glutathione peroxidase and decreased initial phase lipid peroxidation (conjugated dienes and trienes) in a dose dependent manner. Sperm morphology and chromatin condensation were monitored on Feulgen-stained smears. No significant differences were observed between the treatment groups and between sampling time points. The live cell ratio in the sperm (as assessed with flow cytometry) was not different among groups at any of the five sampling timepoints and was also identical within groups. Similarly, the spermatozoa membrane lipid profile was also identical in all three groups after the total intoxication period. In summary, it was demonstrated that FBs in an unrealistic and unjustified high dose still do not exert any drastic harmful effect on the leporine, male reproduction system, meanwhile slightly augmenting testicular antioxidant response.

## 1. Introduction

Fumonisins are mycotoxins (fungal secondary metabolites) produced by *Fusarium verticillioides* and *proliferatum* filamentous fungi (*Liseola* section), mostly infecting cereal commodities, the starch feed basis of monogastric farm animals. The 28 fumonisin analogues characterized since 1988 can be divided into four main groups: series A, B, C, and P [1], from which the B analogues are toxicologically the most hazardous, fumonisin B_1_ (FB_1_) being the most well-known and the most toxic [2]. Fumonisin occurrence is primarily frequent in corn; the prevalence was 78% in 2020 in the samples tested [3]. When considering rabbit feeds, the natural occurrence of fumonisins is relatively low, since corn is rarely exceeding 20% proportion in them; as we are currently not aware of any large scale screening dataset [3], the fumonisin contamination of rabbit feeds might be deduced from the relevant corn content [4].

The harmful effects of FB_1_ are not only species specific in vertebrates (for reviews see: [5,6]), but its mode of action is also approached in detail [6] and provides organ specificity. In brief, FB_1_ is a ceramide conformational analogue and thus a competitive inhibitor of CoA-dependent ceramide synthase [7]. Target organs of FB_1_ are liver and kidney in most domestic animal species; the harmful effect on these organs is exerted via an altered (perturbed) sphingolipid metabolism, leading ultimately to apoptotic and oncotic necrosis, and carcinogenesis in rodents [6]. The consequences of fumonisin-mediated disruption of sphingolipid metabolism are most likely altered cell regulation, since the cellular concentrations of free sphingoid bases are increased and ceramide biosynthesis becomes inhibited. Both above compounds are capable in the induction of cell death, and, according to Riley et al. [6], tumorigenesis is basically initiated by the imbalance between ceramide (↓), sphingosine 1-phosphate (↑), and altered fatty acid (FA) profile [7].

Besides marked contribution to cell disruption, FB_1_ has also been reported to initiate oxidative stress (induction of reactive oxygen species, ROS) in variable cell types, such as neural cell cultures [8] and iliac endothelial cells [9]. Indeed this specific point, the ROS-mediated initiation of the dis-regulation of the cellular membrane permeability [9,10] is a characteristic point of FB_1_ toxicosis that also leads to cell necrosis or swelling. In addition, ROS production and the involvement of the antioxidant system is not limited to defined tissue or cell types, it seems to be a more general event [11], but this has never tested in the male reproduction system (only in one, in vitro approach by Minervini et al. [12] in an equine spermatozoa test).

Meanwhile the nephrotoxic, hepatotoxic, neurotoxic, and carcinogenic impact of FBs is generally well documented [2,7], concerning their in vivo, systematic effects on the male reproductive system and performance there is a relative lack of literature and is mostly limited to rabbits [13,14,15]. Though testicular and epididymal characteristics have been documented in detail, the possible oxidative stress and the accompanying lipid profile modifications in rabbit have not yet been addressed, especially not at high exposure levels. Moreover, in the field of spermatology, mostly quantitative data (volumetric proportions of testicular elements) have been published so far [15], and spermium abnormality types have not yet been tested.

Domestic rabbits have an economical importance within the animal production industry, moreover, rabbits are suggested to be an appropriate model species for reproductive toxicology studies [16].

As far as we are aware, neither oxidative stress, nor dose dependence has been tested in rabbits undergoing fumonisin B series feeding. This targeted study was thus aimed to test the leporine male response on a lower and higher FB dietary level in a (1.) dose dependent manner, in a relatively complex and long approach (to markedly exceed the 49 day-long spermatogenesis time), involving (2.) the testicular histopathology and lipid profile modifications, with (3.) the characterization of the spermatological traits (morphology and composition). The fumonisin dose used in this study was defined to be 2 and 4 times that of the least observed adverse effect level (LOAEL), as established by the European Food Safety Authority [17].

## 2. Results

### 2.1. Animal Performance

**Figure 1:**
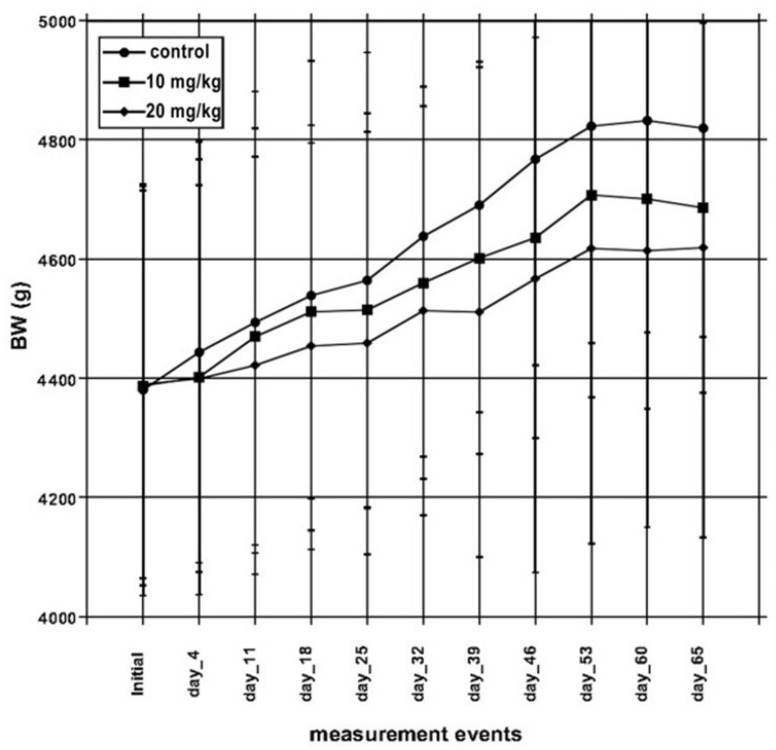
Bodyweight changes along the study period of the three rabbit groups (data points represent group means of each 10 individuals and error bars represent ± SD).

All biological parameters are shown sub-divided according to experimental groups. The initial and final bodyweight (BW) is shown in Table 1 and Figure 1. Since none of the recorded BW group-means differed (Figure 1 and Table 1), BW gain was not considered. As shown in Figure 1, the graded FB levels likewise lowered group mean BW, in a systematic manner, but without statistical significance. The feed intake was measured daily until day 25, then weekly, but provided no inter-group differences (data not shown).

During dissection, the weight of internal organs was recorded. The liver and kidney absolute weights were different between the two intoxicated groups, but spleen and (paired) testis weights (Table 1) were not different among any of them. Relative liver weight showed the same differences like the absolute weight, while other relative organ weights were identical in all three groups.

### 2.2. Spermium Morphology, Chromatin Integrity and Viability

**Figure 2:**
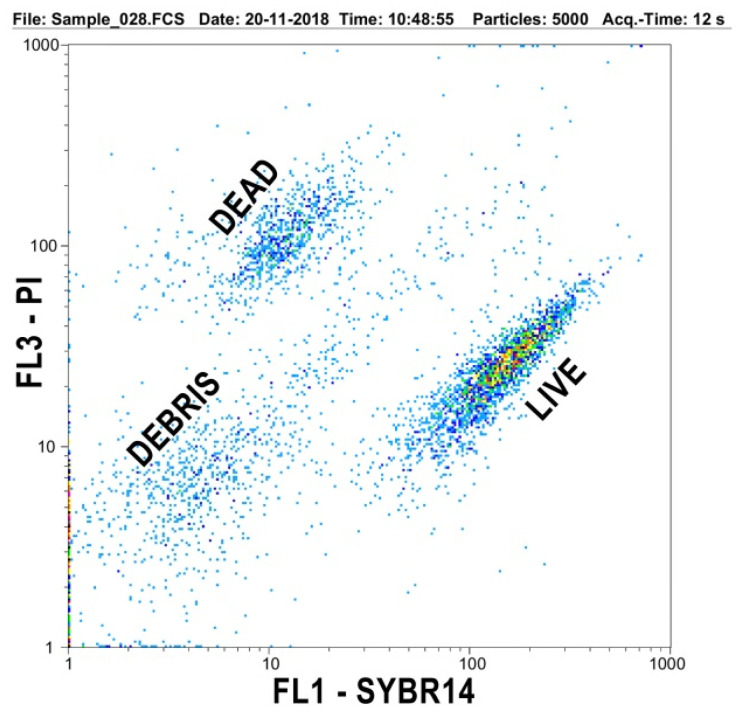
Flow cytometric dot-plot showing the green (SYBR14) and red (PI) fluorescence properties of live and dead spermatozoa; the lower left population was identified as debris and was excluded from data analysis.

**Figure 3:**
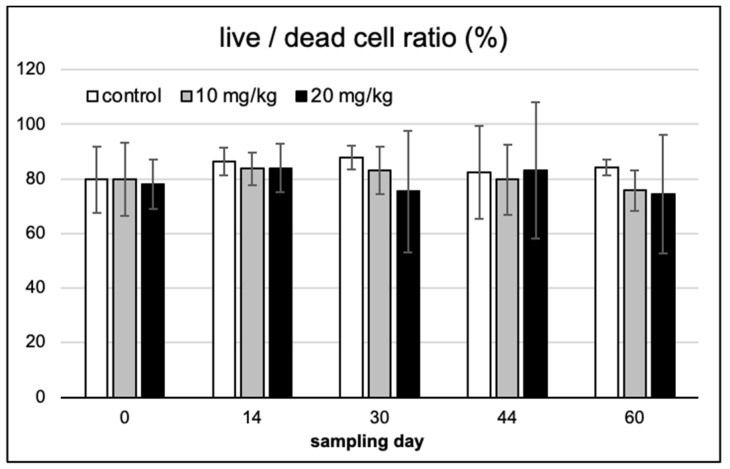
The live cell % data (as assessed from the flow cytometric measurements) of the sperm samples in the five consecutive sampling events, in the three experimental groups (means of 10 individuals ± SD).

The flow cytometric live and dead cellular distribution pattern is shown in Figure 2 in a representative sample, providing a very effective separation. Debris events were excluded from the analysis. When analyzing the live cell proportion within the total cell counts, no systematic difference could be established (Figure 3); differences were not significant even at *p* < 0.05 if analyzing the five consecutive samplings within the single groups and even if the three experimental groups were compared with each other at the five sampling events. Live/dead spermatozoa ratio at the ultimate sampling was 84.3 ± 2.95, 75.8 ± 7.36 and 74.4 ± 21.8, for the control, 10 and 20 mg/kg treatments, respectively.

### 2.3. Spermium Mophology and Distribution

**Figure 4:**
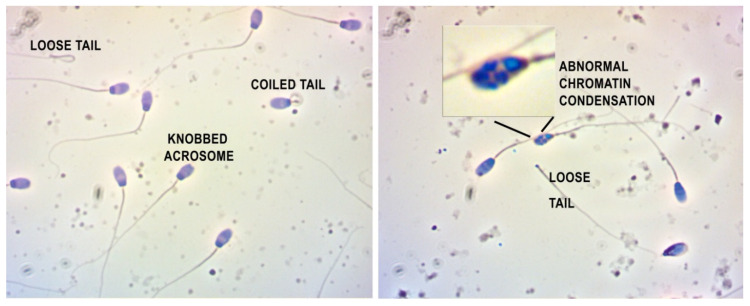
Abnormal morphology of the rabbit spermatozoa. (Feulgen staining, 400× magnification). Inner photo: abnormally condensed chromatin showing patchy staining pattern.

**Figure 5:**
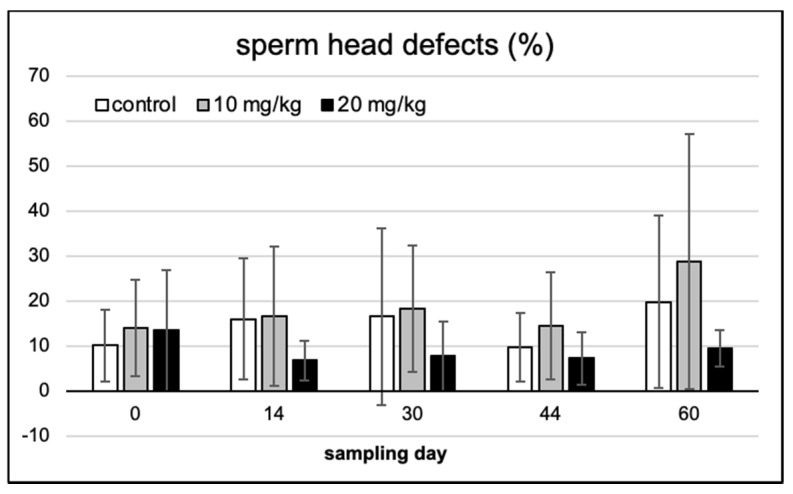
Sperm head defects in the five consecutive sampling events, in the three experimental groups (means of 10 individuals ± SD).

**Figure 6:**
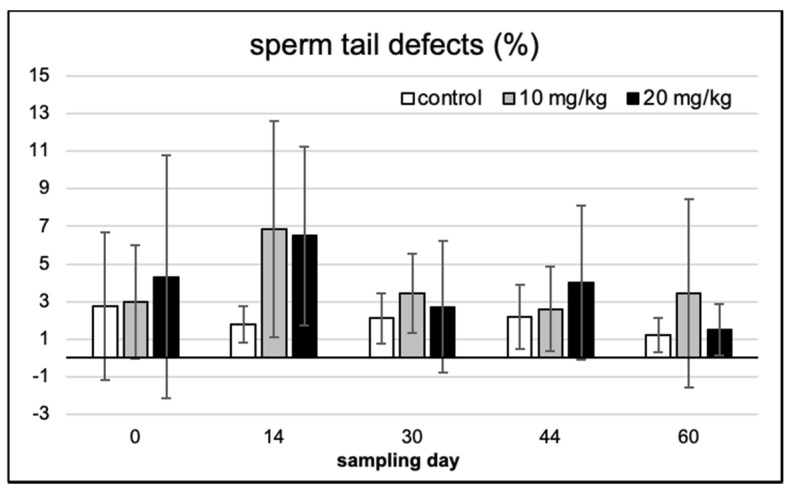
Sperm tail defects in the five consecutive sampling events, in the three experimental groups (means of 10 individuals ± SD).

On Feulgen-stained semen smears, abnormal spermatozoa and disturbances in chromatin condensation were recorded with light microscopic counting. Figure 4 provides the typical demonstrations of some of the most frequent cellular defects observed. Morphological abnormalities were classified as head or tail defect; neither of these showed a significant difference over time and between treatments (Figure 5 and Figure 6).

**Figure 7:**
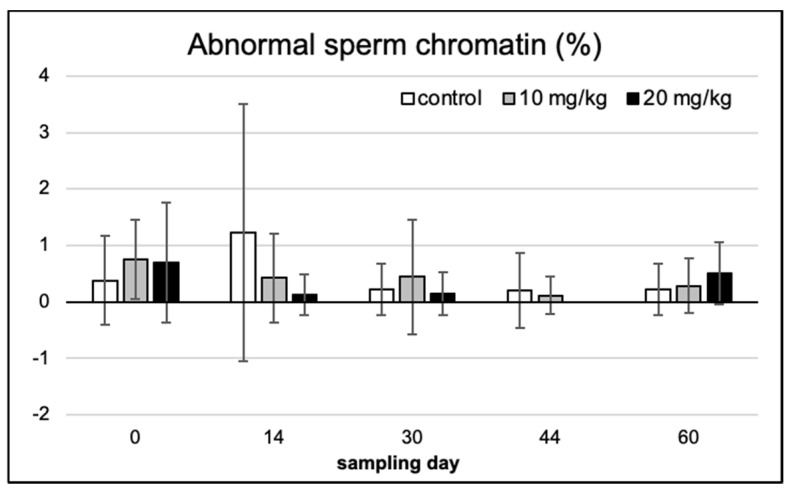
Abnormal sperm chromatin % (occurrence frequency) in the five consecutive sampling events, in the three experimental groups (means of 10 individuals ± SD).

Similarly, no significant effect among the treatment groups or over the exposure time (among sampling days within single treatments) was observed in sperm chromatin condensation (Figure 7).

### 2.4. Testicular Phospholipid Fatty Acid Composition

The testicular total phospholipid fatty acid profile of the three groups is given in Table 2. When comparing the group means, there was a detectable difference only in the proportion of C17:0, margaric acid (↑ in the 20 mg/kg group, *p* = 0.002 for the ANOVA model). Any other individual FAs or calculated variables failed to provide inter-group differences.

Minor further alterations induced by mycotoxin treatment detected were that C14:1 n5, C20:5 n3 and C24:0 were present only in the control samples, while these were absent from the FBs fed animals’ samples.

### 2.5. Testicular Lipid Peroxidation and Antioxidants

**Figure 8:**
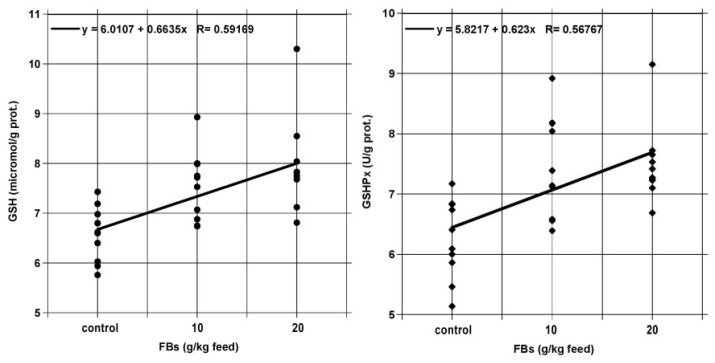
Linear dose-dependence for GSH (●) and GSHPx (♦) in the rabbit testis.

Results of the testicular antioxidant and lipid peroxidation parameters are shown in Table 3. FBs feeding significantly increased the concentration and activity of reduced glutathione (GSH) and glutathione peroxidase (GSHPx), in both intoxicated groups, as compared to the control, in a dose dependent manner (Figure 8). Initial phase lipid peroxidation decreased slightly (CD and CT, i.e. conjugated dienes and trienes) with the increasing FB level, while end-phase lipid peroxidation (MDA) was not proven.

### 2.6. Testis Histology

**Figure 9:**
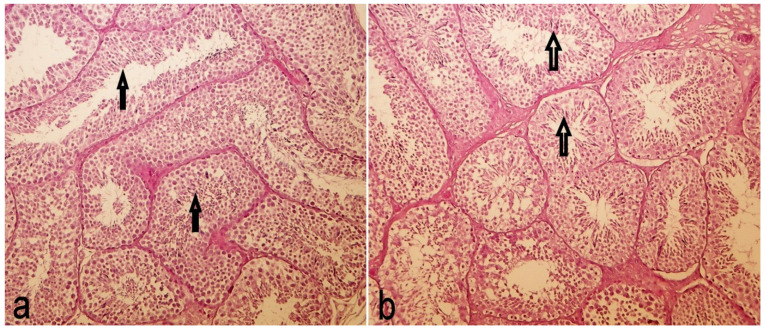
Testis histology of a control (**a**) and an intoxicated (**b**; 20 mg/kg fumonisin Bs, 65 days exposure) rabbit buck (Hematoxilin Eosin staining, 200× magnification).

The testicular tissue sections were evaluated in all animals and provided no group dependent differences. A typical section pair is shown in Figure 9a,b.

Both the cell layers of spermiogenesis and mature spermiocytes (**⇑**) are well visible in the ductuli, without any detectable toxic effect.

### 2.7. Spermium Membrane Fatty Acid Profile

We only determined the control vs. the two intoxicated cases at the last sampling event, and since there was absolutely no systematic inter-group difference detectable, further, retrospective analysis was avoided. Data of the spermium phospholipid fatty acid profile are presented in Table 4.

## 3. Discussion

### 3.1. Animal Performance

The rabbit production performance has also been tested during FB_1_ intoxication in our earlier study, when 10 mg/kg FB_1_ for 4 weeks did not compromise rabbit buck growth [18]. Ewuola (2009) [13] fed rabbit bucks FB_1_ (0.13–5–7.5–10 mg/kg diet) for 196 days and found no throwback in BW. At the same FB_1_ concentration range for 175 days, the onset of puberty was delayed by the two higher doses (i.e., 7.5 and 10 mg/kg), but bodyweight at puberty was not significantly influenced by the toxin. Most probably the exposure time length is the explanation of the unaltered growth, since growth inhibition has already been proven for FB_1_ at 35 mg/kg diet (rats) [19]. The slight, non-significant, but likewise mycotoxin dose associated BW curves in Figure 1 might refer to some growth depression, but without statistical difference, thus it is void to discuss it.

From the splanchnic organs the higher fumonisin dose decreased liver (absolute and relative) and kidney weight; hepatotoxic and nephrotoxic effect of FB_1_ in rabbit is well known [20], but it seems to depend as well on exposure time. In a shorter setting (4 weeks at 10 mg/kg) we found hepatic mitochondrial modifications, but without organ mass difference [18]. In contrast to earlier rabbit testicular results [13,14] with a likewise dose-associated organ weight increase, here we failed to detect any alteration in the testicular weights. We suppose that the reason of this may be that in relevant studies [13,14] growing rabbits were enrolled, meanwhile we started to feed adult, producing males in this study.

### 3.2. Testicular Phospholipid Fatty Acid Composition

The phospholipids (PL) of biological systems are parts of an adaptive, responsive, sensitive domain [21]. We are not aware for any reference data for rabbits in the literature, only Morin (1967) [22] published results for rabbit testicular PLFA. The analysis of whole testicular phospholipids is beyond doubt an approach focusing on multiple cell types; however, this is the lipid fraction that possesses the highest proportion of the polyunsaturated FAs. Testes and spermia show characteristic lipid composition that is rich in longchain polyunsaturated FA (PUFA), primarily DPA (C22:5n3, docosapentaenoic acid) in rats and other rodents [23]. When the channeling of dietary PUFA was checked, it was reported that the primary site of PUFA incorporation is the Sertoli cell population. When exposed to toxic stimulus by FB_1_ (at 7.5 and 10 mg/kg diet for 196 days), Sertoli cell lesion was reported to occur at a moderate level in rabbit testis [12]. Anyway, fumonisin B series induced effect has not yet been tested on the testicular total PLFA profile, especially not at that high exposure level used in this study. Thus, we challenged the animals with a more drastic intoxication, but what we found was negligible.

The only FA that provided a significant proportional alteration was margaric acid (C17:0). This acid is absorbed after coecotrophy into the tissue lipids and is not a product of de novo synthesis. Since the diet did not contain C17:0 in a marked proportion (Table 5), neither feed intake was different among the groups (data analyzed, but not shown), we assume that a change of C17:0 is related to the amount of ingested coecotroph by the host animal, as shown in growing rabbits with the increasing importance of coecotrophy [23,24]. Though we are not aware of the real biological reason, in multiple studies in the past our team detected the significant modification of odd chain FAs (mostly C15:0 and C17:0) in the tissue PL FA profile (rabbit liver ↑, rat liver: C17:0 ↓) [18,25].

The fatty acid profile of testicular phospholipids in germinal and Sertoli cells is a function driven property and is a sensitive indicator of essential FA (EFA) supply or its disturbance [26]. EFA deprivation is reflected in the testicular lipids after 9–14 days [26] and is as well detectable on the cellular accretion of Mead acid (C20:3 n9). We recorded Mead acid proportion (Table 2), but were unable to report dose or group dependent variations, meanwhile present in the tissue PLs in detectable amounts.

In summary, feed intake was identical in the three groups, and feed fatty acid profile (Table 5) was relatively rich in EFA, deficiency was not supposed (Mead acid proportion), meanwhile the overall level of PL unsaturation was relatively low. When EFA deficiency is discussed, not merely the dietary FA supply, but the intensification of lipid peroxidation must as well be considered.

### 3.3. Testicular Lipid Peroxidation and antioxidants

The fumonisin intoxication increased the testicular GSH level, as compared to the control; the increase was not only significant, but provided linear dose dependence (Figure 8). Such a direct oxidative effect of fumonisins has not yet been reported, but heat stress in rabbits has been found to act similarly [27]. According to Aydilek et al. [28], the overall improvement of rabbit antioxidant capacity (e.g., by vitamin E feeding) is accompanied by increased testicular GSH level, and in a more wide context, this effect was shown in case of fumonisin intoxication in porcine liver (20 mg FB_1_/kg diet for 10 days) [29], and at a lower fumonisin level (10 mg/kg dietary FB_1_ for 4 weeks) the opposite was found in the liver of rabbits [18]. Directly relevant mammalian comparison for fumonisin intoxication is unavailable, but at a high and even low dietary FB_1_ dose (600 and 10 mg/kg diet) chicks and broilers provide hepatic oxidative stress [30]. The FBs induced direct oxidative stress is a less studied topic. In contrast, oxidative stress (post-ischemic reperfusion) itself in the leporine testis is a known phenomenon and is accompanied by the increased activity of the enzymatic antioxidant system (GSHPx) [31]. Since testis is a relatively hypoxic microenvironment enriched with a high unsaturated fatty acid proportion [31], its oxidative damage indicators are relatively sensitive.

Currently, there is no full compliance whether fumonisins are direct oxidative stress inducers or this plausible effect is playing a carcinogenic role [32]. Anyway, the molecular mechanisms behind fumonisin toxicity in liver and kidney are supposed to be linked with early events of oxidative stress [33], but the role of them in the male reproductive organs is less studied. As well, a direct link between fumonisins and the glutathione redox system is not fully elucidated; in glutathione peroxidase-1/catalase knocked out (KO) mice FB_1_ toxicity was not influenced by the existence or lack of the induced mutation (KO), referring to a likewise indirect relationship between FB_1_ and oxidative stress [34]. If fumonisin is really not a direct induction factor of the slight oxidative stress detected, then there shall be a plausible alternative process responsible for the dose dependent increase in the tissue GSH level and the associated reaction of GSHPx. Recent and emerging evidence at the molecular level suggests the disruption of mitochondria and excessive generation of toxic, reactive oxygen species as additional mechanisms of toxicity [32]. We assume that quickly proliferating cells (like hepatic and germinal cells) of rabbits may be prone for FBs induced oxidative stress at a subclinical intoxication level, without markedly compromised function. A more general view might be the supposal of slight cellular apoptosis and necrosis, which has only been shown in the renal and hepatic cases [34], but this has not been proven or tested in this study. Ultimately, it shall be added that FBs acted like a slight pro-oxidants in the rabbit testis, augmenting non-enzymatic and enzymatic adaptation (GSH and GSHPx), leading to the lowered concentration of conjugated fatty acid derivatives (dienes and trienes), as early phase lipid peroxidation indicators. This plausible adaptation (exposure: 65 days) was effective, since PLFA profile was unaltered and MDA (as late peroxidation indicator) as well.

### 3.4. Testis Histology

The testicular tissue sections were evaluated in all animals and provided no group dependent differences at all. Intact germinal epithelium, spermiogenesis and mature spermiocytes are well visible in the ductuli, without any detectable toxic effect.

In a prolonged setting [13], the paired testes weight, the seminiferous tubule diameter, and the volumetric proportion of the testicular elements with the exception of the secondary spermatocytes and the Leydig cells provided FB_1_ associated alterations. Though we did not analyze all cell types in such detail as did Ewuola and Egbunike [13,14], we did not find any difference, neither in the paired testicular weight (Table 1), nor in the seminiferous tubule diameter. However, as compared to the data of Ewuola and Egbunike [13,14], the present study enrolled markedly larger bucks and the paired testicular weight was more than two-times higher, as compared to the cited source data. We were unable to detect any patho-morphological signs, while related works [13] refer to depletion of sperm reserves as a mycotoxin effect.

In summary, adult, producing rabbits seem to tolerate relatively high (10 and 20 mg/kg) fumonisin intoxication without detectable testicular patho-morphological alterations.

### 3.5. Spermium Phospholipid Fatty Acid Profile

Fatty acids in male reproduction system are associated with cellular membrane fluidity, acrosome reaction, sperm motility, and viability [5]. Though we did not detect any inter-group differences as possibly evoked by the toxic effect, additionally we found a relatively low level of (poly)unsaturation in the FA profile [35]. Comparing data to those of similarly prepared rabbit spermatozoa PL FAs, our rabbit samples had a higher level of saturated fatty acids, lower mono-unsaturation (11.4 vs. 15.9 %), markedly lower n3 proportion, but higher n6 FA ratio (5.7 vs. 11.3%). Comparing our dataset to a very early report [36], we found more similar data, but still the present n3 FA proportion was lower. The basic difference observed between the two datasets may be based on the nutritional provision of the n3 fatty acids. The dietary FA profile seems to provide basic support that our animals were taking up a low n3 FA amount (Table 5), but their essential fatty acid demand was fulfilled, as shown by the Mead acids constant proportion (Table 2 and Table 4).

When seeking FB_1_-induced spermium compositional or functional modifications, Ewuola and Egbunike [13] reported decreasing sperm mass activity, motility, and live proportion of spermatozoa of the rabbits in parallel with an increase in the dietary FB_1_ concentration. Mice, rats, and rabbits undergo the disruption of sphingolipid metabolism as a result of FB_1_ feeding, but at sub-clinical levels morphologic evidence is lacking [5]. Fumonisin exposure is not limited to the perturbation of the sphingolipid metabolism; it induces imbalances in phosphoglycerolipid and fatty acid metabolism, though never shown for spermia in vivo; additionally, there is only one equine report on an in vitro test [11]. Neither their nor our test results are robust; these suggest that spermia are not sensitive targets of FBs. Though we directly targeted phosphatides, these were found to be absolutely unresponsive on the treatment applied.

The ultimate reason for this may be (1) the low sensitivity against oxidative stimuli, as shaped by the fatty acid profile that has been a result partly of the (2) less unsaturated diet. In addition, we must admit that not only the membrane composition, but (3) other parameters (live cell proportion, sperm morphology, and chromatin status were as well practically unaltered).

## 4. Conclusions

When feeding producing, adult, male rabbit bucks with high fumonisin levels for over a whole testicular cycle, no marked alteration was detected at five sampling timepoints in the spermatozoa endpoints (live cell proportion, morphological distribution, membrane lipid profile) and only minimally increased antioxidant defense was provoked in the testes (without lipid profile or histological modifications). Results refer to minimal pro-oxidant effect of fumonisins on the male reproductive system without marked harmful effect on the tested spermatological traits.

## 5. Materials and Methods

### 5.1. Animals and keeping

Altogether, 3 × 10 Pannon White rabbit bucks were enrolled in the study at the starting age of 24 weeks. The animals were already in production and underwent sperm collection weekly once before the study. Bodyweight (BW) and feed intake (FI) was recorded throughout the experimental period (BW: Table 1 and Figure 1; FI was recorded, but not shown). Feed was offered ad libitum, as well as drinking water from nipple drinkers. Feed (a commercial rabbit buck feed without medication) chemical composition and fatty acid profile is given in Table 5. The somatic data of the three groups before the experiment and at slaughter are given in Table 1. The rabbits were caged individually in a rabbit stable of controlled environment. The photoperiod was natural in the stable (2018 October–November). The study lasted for a total of 65 days and on day 67 animals were euthanized by exsanguination after sedation (Euthanyl-Pentobarbital Sodium, 400 mg/mL, Dechra Veterinary Products, Shrewsbury, UK) and splanchnic organs, testes, and blood were sampled. During the study period, altogether five times, sperm samples were taken for cell integrity and viability analysis. Ejaculate collection was performed with water (37 °C) filled artificial vaginas having a collection tube as an attachment, performed by the caretaker, using a rabbit fur as a phantom. The collection tubes were immediately incubated to 37 °C.

### 5.2. Feed Mycotoxin Contamination

The basic feed was of commercial origin (Table 5). A *Fusarium verticillioides* fungal culture of high FB_1_ concentration (for production details see: [37] culture name: RL 596) was mixed into the ration of the experimental animals, so as to provide a daily FBs (FB_1_+FB_2_+FB_3_) feed concentration of 10 and 20 mg/kg. The mycotoxin concentration of the control and the experimental feed was determined with LC-MS) [38]. The limit of detection (LOD) for FB_1_ was 3 µg/kg. The diet fed to the control group did not contain detectable amounts of FBs (the full absence of deoxinivalenol, zearalenone and T-2 toxin was as well controlled and confirmed).

### 5.3. Evaluation of Sperm Morphology, Chromatin Integrity, and Viability

Fresh semen samples were immediately transferred to the laboratory at 37 °C. The samples were used for the preparation of smears and for differential staining for flow cytometry.

Smears were dried at room temperature and were stained after Feulgen with a staining kit (Merck-Sigma, Schnelldorf, Germany, Cat. No. 1079070001) according to Barth and Oko [39]. The smears were protected with cover plates using Entellan mounting medium (Merck–Sigma Cat. No. 1079600500) and the cell evaluation was based on visual counting (200 cells/smear) on digital images taken at 400× magnification with an Olympus CX-41 (Olympus, Tokyo, Japan) phase contrast microscope equipped with a digital camera.

Flow cytometry was performed with a Molecular Probes Inc. (Eugene, OR, USA) LIVE/DEAD sperm Viability Kit (L-7011) containing SYBR14 and propidium iodide (PI). The staining protocol followed the description of Nagy et al. [40]. In brief, 100 nM SYBR 14 working solution (Component A of the LIVE/DEAD Sperm Viability Kit, diluted 10-fold with dimethyl sulfoxide 10 µl), and 2.4 mM PI stock solution (undiluted Component B of LIVE/DEAD Sperm Viability Kit, 2 µl) were added to 1 mL sperm (extended to approximately 1 × 10^6^/mL in pre-warmed phosphate buffered saline). Samples were incubated at 37 °C for 10 min in darkness.

The samples were transferred immediately after incubation for flow cytometric analysis. A Partec CyFlow Space equipment (Sysmex Partec GmbH, Görlitz, Germany) was operated with the FloMax software (ver. 2.9.), with a two-laser design (20 mW at 488 nm blue solid state laser and a 40 mW at 635 nm red diode laser). The flow speed was 25 µl/sec and acquisitions were stopped after recording 5000 total events. SYBR14 fluorescence (FL) intensity was recorded on detector FL1 (green) while PI fluorescence intensity on FL3 (red), on log scale. Data files were stored in standard FCS file format.

Flow cytometric results were evaluated with the FloMax software (ver. 2.9., Partec GmbH, Görlitz, Germany), and the live/dead cell ratio expressed as % was handled as end result.

### 5.4. Determination of the Testicular and Spermium Phospholipid Fatty Acid Composition

Samples of circa 300 mg raw testicular tissue (after frozen storage at −70 °C) and the feed were homogenized (IKA T25 Digital Ultra Turrax, Staufen, Germany) in 20-fold volume of chloroform:methanol (2:1 v:v) and total lipid content (complex lipids) was extracted [41]. Sperm samples of the last sampling underwent 3× washing in 10-fold volume of phosphate buffered saline, and the washed cells were extracted (to gain complex lipids) as above. Solvents were ultrapure-grade and 0.01% w:v butylated hydroxytoluene was added to prevent fatty acid oxidation. For the separation of lipid fractions, extracted complex lipids were transferred to glass chromatographic columns, containing 300 mg silica gel (230–400 mesh) for 10 mg of total lipids [42]. Neutral lipids were eluted with 10 mL chloroform for the above fat amount, then 15 mL acetone:methanol (9:1 v:v) was added, while 10 mL pure methanol eluted the total phospholipids. This latter fraction was evaporated under a nitrogen stream and was transmethylated with a base-catalyzed NaOCH_3_ method [43]. Fatty acid methyl esters were extracted into 250 μL ultrapure n-hexane for gas chromatography. After a separation on a Phenomenex Zebron ZB-Wax capillary column (30 m × 0.25 mm × 0.25 micrometer film, Phenomenex Inc., Torrance, CA, USA). The chromatographic evaluation was performed with the LabSolutions 5.93 software, using the PostRun module (Shimadzu, Kyoto, Japan) with manual peak integration. Fatty acid composition was expressed as weight % of total FA methyl esters (g FAME/100 g of total FAME). The identification of the FAs was performed based on the retention time of a certified reference material external standard FA mix (Supelco 37 Component FAME Mix, Merck–Sigma Aldrich, CRM47885).

### 5.5. Testicular Antioxidant Status and Lipid Peroxidation

For the determination of lipid peroxidation and antioxidant status, whole tissue samples were stored at −70 °C until analysis. Lipid peroxidation was determined by the quantification of malondialdehyde (MDA) levels with 2-thiobarbituric acid method [44], and the determination of conjugated dienes (CD) and trienes (CT) according to the photometric method of AOAC (1984) [45]. The concentration of reduced glutathione (GSH) was measured by the method of Sedlak and Lindsay [46] and the activity of glutathione peroxidase (GSPHx) according to Lawrence and Burk [47].

### 5.6. Histopathology

Tissue specimens were stored in 10% neutrally buffered formalin and were embedded into paraffin. For light microscopic analysis microtome slides of five micrometer were prepared and stained with hematoxylin-eosin. The main pathological alterations have been described and scored according their extent and severity as follows: 0 = no alteration, 1 = slight/small scale/few, 2 = medium degree/medium scale/medium number, 3 = pronounced/extensive/numerous. The histopathological analysis was performed according to the Act #2011 (03.30) of the Hungarian Ministry of Agriculture and Rural Development and was in accordance with the ethical guidelines of the OECD Good Laboratory Practice for Chemicals [48].

### 5.7. Statistical Analysis

For the comparison of the group means (enzyme activity, initial and final bodyweight, fatty acid profile data within single rows) univariate (FBs concentration as grouping variable) analysis of variance (ANOVA) was used, with the LSD (least significant difference) “post hoc” test for detailed the inter-group differences. The distribution of the different morphologic spermium groups was compared with the chi^2^ probe. Spermatological variables gained from the five consecutive samplings, at each sampling time were compared with ANOVA; time dependent alteration of the different three groups was tested with repeated measures analysis. For all tests significance level was set to *p* ≤ 0.05. IBM SPSS 20 for Windows (2010) [49] was used for the evaluation.

### 5.8. Ethical Issues

The experiments were carried out according to the regulations of the Hungarian Animal Protection Act. The allowance number for the studies was SOI/31/00308-1/2017 (KA2114) (date of approval: 27 March 2017).

## Figures and Tables

**Table 1:** Internal organ (absolute and relative values), initial and final bodyweight values (means ± SD) of the control and intoxicated rabbit groups (*n* = 10/group; BW: bodyweight).

Group	Control				10 mg/kg				20 mg/kg			
Organ Weight (g)	Mean	±	SD		Mean	±	SD		Mean	±	SD	
liver	91.7	±	17.6	ab	96.9	±	13.2	b	80.9	±	11.6	a
kidney	19.8	±	1.92	ab	20.8	±	1.95	b	18.4	±	1.77	a
spleen	1.80	±	0.41		1.58	±	0.47		1.62	±	0.30	
testes	10.3	±	1.68		10.5	±	1.05		9.6	±	1.58	
rel. liver (%)	1.90	±	0.26	ab	2.07	±	0.29	b	1.75	±	0.17	a
rel. kidney (%)	0.41	±	0.04		0.44	±	0.04		0.40	±	0.04	
rel. spleen (%)	0.04	±	0.01		0.03	±	0.01		0.04	±	0.01	
rel. testes (%)	0.22	±	0.04		0.22	±	0.02		0.21	±	0.03	
initial BW (g)	4380	±	345		4387	±	335		4390	±	325	
final BW (g)	4819	±	350		4686	±	309		4619	±	486	

a,b: different superscripts indicate significant difference between group means at *p* < 0.05.

**Table 2:** The fatty acid (FA) composition (weight % of total FA methyl esters) of the testicular total phospholipids in the three experimental groups (means of 10 individuals± SD).

Group	Control				10 mg/kg				20 mg/kg			
Fatty Acid	Mean		SD		Mean		SD		Mean		SD	
C14:0	0.39	±	0.52		0.31	±	0.15		0.26	±	0.12	
C14:1n5	0.02	±	0.00		nd				nd			
C15:0	0.31	±	0.64		0.10	±	0.01		0.11	±	0.02	
C16:0	35.3	±	3.01		37.0	±	2.32		35.3	±	1.39	
C16:1n7	0.20	±	0.07		0.23	±	0.05		0.21	±	0.03	
C17:0	0.32	±	0.08	a	0.40	±	0.09	a	0.51	±	0.12	b
C18:0	29.0	±	8.17		25.0	±	4.82		24.6	±	3.16	
C18:1n9c	7.95	±	2.45		8.79	±	1.38		9.04	±	0.85	
C18:1n7	0.83	±	0.25		0.96	±	0.15		0.88	±	0.12	
C18:2n6	4.91	±	1.24		5.62	±	0.99		5.47	±	0.76	
C18:3n6	0.12	±	0.05		0.15	±	0.02		0.15	±	0.04	
C18:3n3	0.02	±	0.01		0.08	±	0.18		0.02	±	0.00	
C18:4n3	0.43	±	0.45		0.27	±	0.27		0.30	±	0.14	
C20:0	0.21	±	0.12		0.14	±	0.05		0.14	±	0.03	
C20:1n9	0.13	±	0.04		0.13	±	0.03		0.11	±	0.02	
C20:2n6	0.30	±	0.08		0.35	±	0.08		0.30	±	0.03	
C20:3n9 (Mead acid)	0.09	±	0.03		0.08	±	0.01		0.08	±	0.02	
C20:3n6	7.58	±	2.09		8.68	±	1.78		8.83	±	1.19	
C20:4n6	11.6	±	3.27		11.5	±	2.30		13.3	±	1.48	
C20:5n3	0.10	±	0.03		0.09	±	0.01		nd			
C22:0	0.06	±	0.06		0.03	±	0.01		0.03	±	0.01	
C22:5n3	0.18	±	0.07		0.15	±	0.06		0.18	±	0.04	
C24:0	0.13	±	0.10		nd				nd			
C22:6n3	0.12	±	0.05		0.11	±	0.03		0.13	±	0.06	
Saturated	65.6	±	9.27		62.9	±	6.04		61.0	±	3.73	
Unsaturated	34.4	±	9.27		37.1	±	6.04		39.0	±	3.73	
Monounsaturated	9.09	±	2.80		10.1	±	1.55		10.2	±	0.97	
Polyunsaturated	25.3	±	6.60		26.9	±	4.59		28.8	±	2.83	
n3	0.77	±	0.48		0.58	±	0.33		0.62	±	0.14	
n6	24.5	±	6.36		26.3	±	4.47		28.1	±	2.79	
n6/n3	39.5	±	18.5		64.5	±	43.0		46.7	±	9.80	
Odd chain FA	0.61	±	0.59		0.49	±	0.10		0.61	±	0.13	
Unsaturation index	93.6	±	24.9		98.1	±	16.7		106.1	±	10.5	
Average FA chain length	17.68	±	0.13		17.67	±	0.11		17.74	±	0.07	

a,b: different superscripts indicate significant difference between groups means at *p* < 0.05; nd: not detected.

**Table 3:** Testicular antioxidant and lipid peroxidation parameters in the three experimental rabbit groups (GSH: reduced glutathione; GSHPx: glutathione peroxidase; MDA: malondialdehyde; CD: conjugated dienes; CT: conjugated trienes, U: unit, A: absorbance).

Group	Control				10 mg/kg				20 mg/kg			
GSH (micromol/g prot.)	6.57	±	0.55	a	7.54	±	0.70	b	7.90	±	0.98	b
GSHPx (U/g prot.)	6.25	±	0.66	a	7.45	±	0.85	b	7.50	±	0.65	b
MDA (nmol/g)	44.7	±	9.11		39.0	±	7.17		38.5	±	7.22	
CD (A232 nm)	0.34	±	0.02	b	0.34	±	0.03	b	0.31	±	0.02	a
CT (A268 nm)	0.16	±	0.01	b	0.15	±	0.01	ab	0.14	±	0.01	a

a,b: different superscripts indicate significant difference between groups means at *p* < 0.05.

**Table 4:** Phospholipid fatty acid (FA) composition (weight % of total FA methyl esters) of the washed spermia after 65 days of FBs exposure of rabbit bucks to 0, 10, and 20 mg/kg diet FBs (n.d.: not detected).

Group	Control			10 mg/kg			20 mg/kg		
	Mean		SD	Mean		SD	Mean		SD
C14:0	2.61	±	0.50	2.32	±	0.57	2.57	±	0.92
C15:0	0.19	±	0.07	0.49	±	0.34	0.46	±	0.35
C16:0	25.0	±	0.28	26.3	±	1.05	26.4	±	0.98
C16:1	0.68	±	0.37	0.80	±	0.12	0.78	±	0.61
C17:0	0.74	±	0.49	1.13	±	0.61	1.04	±	0.12
C17:1n7	0.18	±	0.03	0.24	±	0.12	0.28	±	0.14
C18:0	47.5	±	3.76	39.0	±	9.26	41.1	±	6.85
C18:1n9	9.62	±	3.41	14.9	±	6.85	13.3	±	6.96
C18:1n7	0.61	±	0.06	0.82	±	0.33	0.97	±	0.44
C18:2n6	5.01	±	1.98	8.54	±	3.90	7.16	±	2.45
C18:3n6	0.02	±	0.01	0.09	±	0.06	0.06	±	0.03
C18:3n3	0.10	±	0.01	0.08	±	0.07	0.05	±	0.02
C20:0	n.d.			0.15	±	0.03	0.17	±	0.15
C20:1n9	0.08	±	0.02	0.08	±	0.05	0.02	±	0.01
C20:3n9 (Mead acid)	0.31	±	0.05	0.26	±	0.15	0.28	±	0.19
C20:3n6	3.18	±	0.80	2.21	±	1.14	2.64	±	1.28
C20:4n6	3.11	±	0.93	1.80	±	0.91	1.93	±	1.05
C20:5n3	0.28	±	0.01	0.21	±	0.07	0.17	±	0.05
C22:0	0.11	±	0.01	n.d.			0.05	±	0.04
C22:1n9	0.08	±	0.01	n.d.			0.07	±	0.01
C24:0	0.41	±	0.01	0.11	±	0.06	n.d.		
C22:6n3	0.37	±	0.02	0.29	±	0.18	0.34	±	0.11
C24:1n9	0.19	±	0.16	0.19	±	0.07	0.14	±	0.13
Saturated	76.4	±	4.17	69.6	±	8.99	71.9	±	8.23
Unsaturated	23.6	±	4.17	30.4	±	8.99	28.1	±	8.23
Monounsaturated	11.4	±	4.00	17.0	±	7.34	15.5	±	8.30
Polyunsaturated	12.2	±	0.17	13.4	±	1.88	12.6	±	0.07
n3	0.56	±	0.14	0.51	±	0.24	0.56	±	0.04
n6	11.3	±	0.26	12.6	±	2.18	11.8	±	0.15
n6/n3	20.8	±	5.59	37.4	±	36.4	21.1	±	1.62
Odd chain FA	0.93	±	0.56	1.62	±	0.93	1.50	±	0.23
Unsaturation index	47.4	±	1.15	51.7	±	7.28	49.5	±	4.37
Average FA chain length	17.54	±	0.01	17.45	±	0.04	17.45	±	0.01

**Table 5:** Chemical and fatty acid composition of the basal diet of the experimental rabbits.

Chemical Composition		Fatty Acid (Diet)	Weight % of Total FAME
Dry material (%)	89.0	C12:0	0.05
Crude protein (%)	14.5	C14:0	0.18
Ether extract (%)	2.4	C15:0	0.14
Crude fibre (%)	17.1	C16:0	14.3
Ash (%)	7.5	C16:1n7	0.20
Lysine (%)	0.90	C17:0	0.12
Methionine (%)	0.41	C18:0	2.73
Calcium (%)	0.88	C18:1n9	36.8
Phosphorus (%)	0.52	C18:1n7	0.84
Sodium (%)	0.19	C18:2n6	38.6
Vitamin A (IU/kg)	14000	C18:3n3	3.83
Vitamin D3 (IU/kg)	1300	C20:0	0.42
Vitamin E (mg/kg)	107	C20:1n9	0.50
Digestible energy (MJ/kg)	9.7	C20:2n6	0.04
		C21:0	0.04
		C20:4n6	0.07
		C22:0	0.61
		C24:0	0.45
		C22:6n3	0.08
		Saturated	19.0
		Unsaturated	80.9
		Monounsaturated	38.3
		Polyunsaturated	42.6
		n3	3.91
		n6	38.7
		n6/n3	9.89
		Odd chain FA	0.26
		Unsaturation index	127.8
		Average FA chain length	17.75

## Data Availability

Data available on request due to restrictions e.g., privacy or ethical (The data presented in this study are available on request from the corresponding author. The data are not publicly available due to [large dataset and data are not self explanatory].).
